# The Strategic Advantage of FQHCs in Implementing Mobile Health Units: Lessons Learned from a Pilot Initiative

**DOI:** 10.3390/ijerph23020158

**Published:** 2026-01-27

**Authors:** Lauren Bifulco, Anna Rogers, Cecilia Hackerson, Marwan S. Haddad, April Joy Damian, Kathleen Harding

**Affiliations:** 1 Weitzman Institute, Moses/Weitzman Health System, Middletown, CT 06457, USA; bifulcl@mwhs1.com (L.B.); haddadm@chc1.com (M.S.H.); damiana@mwhs1.com (A.J.D.); 2 Center for Key Populations, Community Health Center, Inc., Moses/Weitzman Health System, Middletown, CT 06457, USA; rogersa@chc1.com (A.R.); hackerc@chc1.com (C.H.); 3 Department of Mental Health, Johns Hopkins Bloomberg School of Public Health, Baltimore, MD 21205, USA

**Keywords:** mobile health unit, community health centers, primary care, healthcare delivery, healthcare access, community-clinical linkage

## Abstract

**Highlights:**

**Public health relevance—How does this work relate to a public health issue?**

- Federally Qualified Health Centers (FQHCs) are community-based and community-led organizations in the United States that receive federal funds to provide comprehensive primary care and support services to community members in underserved areas whose social, economic, environmental, and physical needs may present barriers to accessing and obtaining quality care.
- Mobile Health Units (MHUs) are community-based points of care that can help FQHCs reach populations for whom traditional site-based care remains inaccessible due to systematic barriers and who may not engage with the healthcare system otherwise.

**Public health significance—Why is this work of significance to public health?**

- This descriptive observational implementation study presents initial outcomes of a new MHU program implemented by a large, statewide FQHC system and offers recommendations for FQHCs and similar organizations that are starting a new or expanding an existing MHU program.
- Based on the firsthand experience of our MHU program’s providers, care teams, and program leaders, we offer specific recommendations and lessons learned regarding patient engagement and community connection, MHU logistics, addressing healthcare access needs, and addressing transportation as a barrier.

**Public health implications—What are the key implications or messages for practitioners, policy makers and/or researchers in public health?**

- The FQHC-led MHU model is a potentially scalable solution for community health centers and other organizations interested in providing patient-centered and community-centered delivery of healthcare services and health-related knowledge to community members.
- Allocating resources to mobile primary care delivery to hard-to-reach populations enhanced our FQHC’s ability to offer proactive, patient-centered care, with the intent of strengthening patients’ engagement with high-quality community-focused primary care.

**Abstract:**

High-need populations face substantive barriers to accessing primary care, leading to disproportionately poor health outcomes. This descriptive, observational study details the implementation of a Federally Qualified Health Center (FQHC) program designed to improve engagement in care and enabling services by leveraging mobile health units (MHUs) to provide comprehensive, low-barrier primary care services to residents who were previously unable or unwilling to engage with the traditional healthcare system. The program sought to overcome common access challenges such as lack of transportation, lack of insurance, and mistrust of healthcare institutions. We describe the operational framework of this program, examine the types of care delivered, and offer recommendations from the perspective of a large multi-site FQHC experienced in reengaging people back to the healthcare system but new to providing mobile health care. We describe our program’s focus on prioritizing patient engagement and access and its consideration of operational and technical infrastructure. Based on our FQHC’s experience, we provide recommendations on how to address patients’ health and social needs. FQHCs have the potential to implement MHUs, drawing on their existing infrastructure and community relationships. Our MHU program is well-aligned with our FQHC’s commitment and priority to deliver essential care and foster continuity within hard-to-reach communities, strengthening the local healthcare safety net and improving healthcare for high-need populations.

## 1. Introduction

It is estimated that 75 million people in the United States (U.S.) live in primary care health professional shortage areas, where the population-to-provider ratio exceeds 3500:1, and that 7.3% of U.S. counties have no primary care physicians [1,2]. Approximately 100 million people live in communities that experience barriers to healthcare access [3], 15.6 million live in households with income below the federal poverty level, and one in five (20.6%) experience three or more indicators of social need, such as unemployment, disability, and food insecurity [4].

There are approximately 3000 mobile health clinics in the U.S., which generate 10 million visits per year [5]. Mobile health clinics provide clinical-community linkages that bridge gaps in medical services for people with limited access [6]. They serve geographically isolated, rural, and other areas with sparse access to care, and help connect people in these areas back to the health care system. These clinics leverage mobile health units (MHUs) to bring health care and other services directly to those with difficulty accessing care, which helps overcome barriers to care including lack of transportation, distance, and inconvenience [7]. MHUs are ideal for providing patient-centered and community-centered delivery of healthcare services and health-related knowledge to community members who may not engage with the healthcare system otherwise.

Primary care delivered to uninsured, underinsured, and publicly insured people in safety-net settings reaches people whose social, economic, environmental, and physical needs may present barriers to accessing and obtaining quality care [8,9]. Despite the increasing presence of MHUs in the U.S., few studies have detailed their optimal use and impact when integrated into a community-based healthcare system. Federally qualified health centers (FQHCs) are community-based and community-led organizations that receive federal funds to offer a range of comprehensive primary care and support services designed to meet the needs of community members [10]. In the state of Connecticut, FQHCs serve approximately 450,000 people (one in eight residents), regardless of insurance status or ability to pay for care [11]. In contrast with retail clinics, urgent care centers, emergency departments and similar sources of on-demand care that many uninsured and publicly insured residents rely on, FQHCs offer primary care services which provide the opportunity for a patient to develop a long-term relationship with a provider and care team who can help them coordinate care that will meet their health needs going forward [10,12]. The objective of this observational study is to describe the reach of an MHU program serving populations located in and near the catchment area of a statewide multisite FQHC system in Connecticut which is the primary care medical home for approximately 110,000 people.

We address a gap in the literature on integrating MHUs into FQHC-based healthcare, and describe our experience rapidly standing up mobile health clinic services that offer low-barrier primary care treatment. We present information on how we extended the FQHC’s service offerings using MHUs and share recommendations and lessons learned when expanding mobile health service offerings to multiple locations statewide.

## 2. Materials and Methods

### 2.1. Participants and Setting

Community Health Center, Inc. (CHCI) is a statewide multi-site FQHC network in Connecticut (CT) with 15 fixed sites and 200+ touchpoints throughout the state. Its Center for Key Populations (CKP) serves community members who experience significant barriers to comprehensive, respectful, and safe healthcare, including those with serious health challenges such as housing insecurity and homelessness, substance use disorders, HIV, viral hepatitis, and domestic violence. Many of the CKP’s patients struggle to access care in traditional settings, exacerbated by financial, psychosocial, and transportation challenges. CKP’s Wherever You Are Healthcare for the Homeless program was established in 2005, and offers medical, dental, and behavioral health care services at seven emergency shelters and mental health, substance use, and community-based organizations in five towns statewide. An additional farmworker health program offers monthly medical, behavioral health, oral health, and substance use disorder service clinics for approximately 500 workers per year at 25 farms statewide.

While delivering care to high-need populations statewide in response to the COVID-19 pandemic, CHCI/CKP identified groups of patients who were not engaged with the health care system. In February 2023, in direct response to patient demand, CKP purchased its first 26-foot MHU, which was initially staffed by a core team consisting of a primary care provider (nurse practitioner) accompanied by a driver/community health worker, which served patients in three towns (Bristol, Middletown, and Wallingford, CT). After acquiring a second MHU we established primary care MHU services in Hartford, CT (9/2024; men’s health), Meriden, CT (10/2024), and Windham County, CT (5/2025; maternal and women’s healthcare).

### 2.2. Data Collection and Analysis

We assessed the reach of our program by analyzing barriers to care prior to MHU service delivery and service utilization. To better understand barriers to care prior to MHU service delivery, we conducted a secondary analysis of existing information from (1) key stakeholder analysis activities completed prior to initiating MHU service delivery in each new location, and (2) program planning documents, operational reports, and summaries of meetings with MHU program leadership and staff. Key stakeholder analysis data sources included summaries and qualitative memos of group and individual discussions and community meetings led by CKP. Question guides were developed by MHU programmatic leadership and team members in order to obtain the perspectives of convenience samples of patients, potential patients, community partners, and the public on gaps in healthcare services and access. Qualitative data were analyzed in Microsoft Excel (Microsoft 365 Apps) using inductive (bottom up) thematic analysis [13]. The first author, who was not present during the stakeholder analysis activities, conducted initial review and assignment of data elements to themes, and led iterative review and discussion of themes with MHU programmatic leadership and staff until consensus was reached. Team members who contributed to thematic analysis included the program director (who had led group, individual, and community discussions during the stakeholder analysis process), two primary care providers who treat patients on the MHU, and the project director for the maternal health MHU program.

We analyzed quantitative service utilization data (patient demographics, visits provided, unique patients served, services provided) retrieved from the electronic health record at CHCI. Quantitative data were analyzed in Microsoft Excel using descriptive statistics (counts, measures of central tendency) in order to summarize the characteristics of patients who used the MHU and services provided. This retrospective secondary analysis of existing programmatic data was determined by the Institutional Review Board at Community Health Center, Inc. to be exempt from full review (Protocol #1238, 30 October 2025).

## 3. Results

### 3.1. Patients Served

Between March 2023 and October 2025, CKP’s MHU program provided 1298 visits and served 581 unique patients. Of these, 183 (31.5%) returned to the MHU once or more following their initial visit (average: 2.2 visits per patient, standard deviation: 3.2, range: 1–23). Table 1 shows the characteristics of patients served, common reasons for seeking care, and common types of enabling services sought by community members.

### 3.2. Barriers to Care Prior to MHU Service Delivery

Community needs prior to MHU service delivery coalesced around environmental, social, physical, and economic barriers to seeking care. Patients described the inconvenience of attending appointments at a specific place and time, difficulty with physical distance to fixed sites, feelings of stigma and fear, and difficulty obtaining or affording needed services. Based on our analysis, the barriers patients encountered when seeking care in traditional settings such as fixed healthcare sites included: lack of accessible hours and walk-in appointments, lack of access to providers who understood their specific health-related and social needs, lack of consistent access to the same provider, challenges related to physical distance and lack of transportation, and lack of high-quality and affordable primary care, behavioral health, and enabling services.

**Figure 1:**
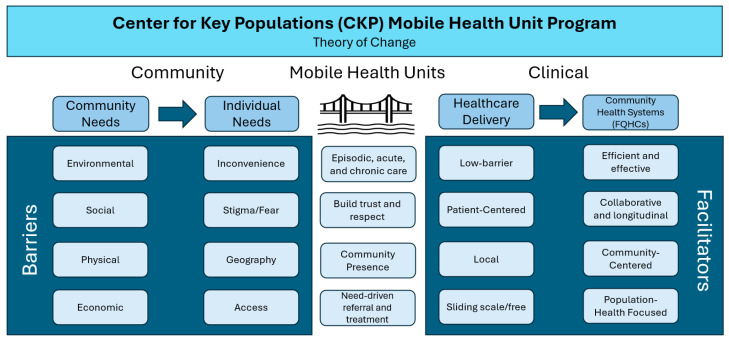
Using Mobile Health Units to Link Patients to High-Quality Primary Care. Our program introduced mobile health units (MHUs) to bridge gaps imposed by community and individual barriers to seeking care, with the intent of strengthening patients’ engagement with high-quality community-focused primary care, as is offered in federally qualified health centers (FQHCs).

Figure 1 shows our program’s strategic vision for addressing patients’ barriers to care by using MHUs to engage them with the healthcare system in a manner that anticipates and addresses their needs.

### 3.3. Recommendations for Policy and Practice and Lessons Learned

Based on our firsthand experience, we recommend that FQHCs adding MHUs to their scope dedicate their initial attention and resources to patient engagement and community connection, logistics, and consideration of how best to address healthcare access needs. Table 2 provides recommendations for policy and practice regarding initial implementation of an MHU by an FQHC.

#### 3.3.1. Patient Engagement and Community Connection

Prioritizing patient engagement and community connection before beginning service delivery helps demonstrate the MHU’s ability to provide consistent, high-quality services and lays the groundwork for patients to engage more consistently in routine and preventive care (Table 2).

#### 3.3.2. Logistics

Our MHU program aimed to provide the same services offered at fixed sites, which required integration with the FQHC’s existing electronic data capture infrastructure, including its patient registration and electronic medical record systems. Please refer to Table 2 for strategic recommendations on use of the electronic health record and electronic scheduling, operational oversight, and forming an MHU team.

#### 3.3.3. Addressing Healthcare Access Needs

By committing to a patient-centered model defined by accessibility, consistency, and a robust operational foundation, an MHU can serve as a powerful tool for delivering high-quality health services. FQHCs implementing MHU services can anticipate the need to consistently reinforce their support for the priorities of the communities they serve, particularly with respect to enabling services, social setting, trust, and consistent care. (Table 2).

#### 3.3.4. Addressing Transportation as a Barrier

When establishing MHU services in a new location, it is particularly important to assess the community’s transportation needs and proximity to available services, and proactively plan to address challenges. We deploy a separate minivan alongside the MHU for its team members (e.g., the MHU driver) to transport patients and ensure that they are able to obtain the lab tests their provider orders as well as pick up medications from the pharmacy. We have made local laboratory testing facilities aware of MHU service dates in order to help anticipate increased demand for testing, and improve efficiency for patients and the facility. We also worked with local pharmacies who are willing to deliver prescribed medications. 

Having transportation available onsite helps mitigate two key barriers to providing high-quality care on the MHU: obtaining relevant laboratory and diagnostic data, and ensuring timely access to medications for patients. Many individuals seen on the MHU are new patients, and their medical records are often unavailable during their initial visit. The ability to obtain timely laboratory results allows providers to diagnose and manage medical conditions more safely and efficiently, reducing the risk of undiagnosed or untreated health issues in this population. Additionally, having transportation onsite enables patients to pick up medications from pharmacies on the same day, facilitating medication adherence and allowing providers to initiate and adjust treatments more quickly and effectively.

Our preliminary data indicate that these methods are working. Before we made extended transportation resources available, approximately 16.7% (11 of 66) of lab requests were completed and 37.0% of patients (27 of 73) with prescriptions from an MHU provider had obtained them, whereas approximately 97.8% of lab requests (91 of 93) and 94.2% of prescriptions were obtained afterwards (98 of 104). 

Our maternal health MHU is piloting a different approach, using dedicated grant funds to cover a rideshare service account (Uber Health), which anyone on the MHU team can use to arrange patient transportation to local lab testing service locations. We anticipate similar capacity to increase uptake of recommended laboratory testing and prescriptions.

## 4. Discussion

The deployment of an MHU is a potential strategy for FQHCs to engage hard-to-reach populations and use episodic and acute care to lay the foundation for the patient to establish routine primary care. Our MHU program provided care for 581 patients, and one in three returned to the MHU for services. Patient-focused, low-barrier service delivery can contribute to mitigating the impact of distrust in the healthcare system as a barrier to healthcare access and engagement [7]. This descriptive observational study provides an example from a large statewide FQHC system that has integrated an MHU program into its FQHC service offerings.

FQHC primary care provides broad, comprehensive, overlapping health care services that bridge prevention, wellness, and treatment [14]. Primary care providers offer continuity of care for both simple symptoms and complex, interdependent health needs and fulfill patients’ social and emotional needs for trust, respect, and advocacy [15]. Having a usual source of care such as a primary care provider promotes receipt of preventive healthcare and is associated with increased treatment compliance, decreased morbidity, and increased life expectancy [14,16]. In addition to meeting the immediate need of a patient who may use the MHU services once or who may only want to receive services on the MHU, our mobile health unit program is able to connect patients back to the primary care system, which can also serve as a conduit to connecting patients to specialty and subspecialty services when needed—for example, through asynchronous electronic specialty consultation [17] or direct referral—both of which are available to help primary care providers on our MHUs obtain consultation, feedback, and treatment recommendations for the patients they treat.

It is anticipated that FQHCs and other community health centers will play an increased role in caring for a projected 1.9–4.2 million additional uninsured people by 2034, despite potential gaps in state and federal funding and difficulty filling vacant frontline care team roles [18]. MHUs can increase the quality of care delivery and help remove impediments to good physical and mental health, with the goal of improving quality of life by improving health outcomes. The MHU brings the care environment to the patients and encourages shared decision-making, patient-directed care, and self-determined goal-setting, which are foundational to building trust between patients with complex care needs and their healthcare providers [19]. CKP’s MHU program was designed to address significant barriers to care such as access challenges, lack of trust in traditional health care settings, and the need for flexible, responsive services, and offers a safe point of entry into care that welcomes patients and meets them where they are.

Our firsthand experiences are valuable for FQHC clinical and administrative leaders and research personnel who are interested in the logistics and initial outcomes of a new MHU program. We provide perspective on the experience of standing up a functional MHU in several different locations and settings and developing and executing a plan for its operation. We offer initial data on services provided and recommended implementation strategies, which are crucial for program planning.

Workforce recruitment and retention is a sustainability challenge for MHU programs [20]. A strength of our program is that our MHUs are staffed by primary care providers with postgraduate nurse practitioner residency and fellowship training in caring for key populations like those served by our MHU [21], and that our program was developed and implemented by a health system that has already built mutual trust and respect with local communities, and helmed by the clinical and administrative leaders of a dedicated center committed to improving access and quality of care for its patients. These attributes of our program may limit transferability to other settings. Though our FQHC possesses the clinical expertise, administrative systems, and experience in patient care that are crucial for establishing and maintaining an MHU program as part of health center operations, our program’s future financial sustainability is reliant on maintaining and increasing patient volume or pursuing outside funding, a challenge shared by many MHU programs in the U.S. [22]. A detailed financial analysis was out of scope of the present study, which may limit the generalizability of our recommendations and findings to programs implemented outside of FQHC service delivery.

This descriptive, observational study is not generalizable to all MHU programs. Our study is further limited by its short duration, small sample size, and focus on a limited geographic area, which illustrates a central conflict in providing MHU services in a fee-for-service healthcare environment. FQHC leadership and program planners must balance MHUs’ ability to access hard-to-reach populations with practical considerations such as staffing and start-up costs, sustainability, alignment with organizational priorities and capabilities, and the need for time to ramp up services in order to be financially sustainable. 

The majority of studies on MHUs are descriptive or observational [23,24]. The observational nature of our study limits our ability to connect outcomes and context, and we are unable to make causal inferences. Though our observations were specific to a single statewide FQHC, our MHU program serves various, diverse communities in both small and large towns, rural and urban, which include individuals living in shelters, people living in camps in the woods, and communities in parts of the state with healthcare deserts. Thus, we believe that the recommendations we are sharing may be translatable to similar populations served by other FQHCs. Further study will consider the impact of established MHUs over a longer follow-up period, and will incorporate an economic analysis or other cost-effectiveness information. Future study should also examine whether and how this model can be replicated or scaled to additional communities and broader patient populations.

## 5. Conclusions

Our MHU program allowed our FQHC to extend healthcare services to additional patients, complementing our efforts to address the unmet need for primary care in our local communities. We present an assessment of the reach of our program, which included key stakeholder analysis and review of utilization data, and resulting recommendations for policy and practice. We recommend that FQHCs and other organizations building new MHU programs prioritize patient engagement, make mindful decisions about operational and technological infrastructure, and attempt to identify and address barriers to healthcare access. As trusted community organizations, FQHCs are a suitable settings to make an MHU program successful.

## Figures and Tables

**Table 1:** Patients Served and Services Provided (N = 581 unique patients, 1298 visits).

Characteristic	#	%
Age (N = 581)		
18–29	64	11.0
30–39	122	21.0
40–49	132	22.7
50–59	132	22.7
60–69	95	16.4
70+	36	6.2
Gender (N = 581)		
Female	173	29.8
Male	408	70.2
Ethnicity (N = 581)		
Hispanic	253	43.6
Not Hispanic	215	37.0
Unreported	113	19.4
Race (N = 581)		
American Indian or Alaska Native	3	0.5
Asian or Pacific Islander	4	0.7
Black or African American	111	19.1
White	178	30.7
More than one	2	0.3
Other or Unknown	283	48.7
Visit Type (N = 1298 visits)		
Medical	1202	92.6
Nursing	64	4.9
Women’s Health	32	2.5
Most Common Clinical Assessments (by ICD-10 code; 1298 visits) ^1^		
HIV, Viral hepatitis, and STI screening	495	38.1
Blood Pressure	321	24.7
Pain	295	22.7
Substance Use, Including Tobacco and Alcohol Use	294	22.7
Mental Health	267	20.6
Overweight or Obesity	259	20.0
Diabetes and Pre-Diabetes, including Screening	255	19.6
Maternal and Women’s Healthcare MHU Navigation Services (n = 29 patients) ^2^		
Access/Engagement in Healthcare—Primary Care	16	55.2
Medical Transportation	14	48.3
Health-Related Social Need—Housing	11	37.9
Insurance Navigation	10	34.5
Health Risk—Connecticut Early Cancer Detection and Prevention Program	7	24.1

^1^ Each patient could have had multiple reasons for visit. ^2^ Each patient could have received multiple navigation services. Abbreviations: MHU: Mobile Health Unit; ICD-10 International Classification of Diseases, 10th revision; HIV: Human Immunodeficiency Virus; STI: Sexually Transmitted Infection.

**Table 2:** Recommendations for Initial Implementation of an MHU by an FQHC.

Category	Recommendation
Patient Engagement and Community Connection	(a) Work with community partners in places where the need for MHU services exists to establish where and when to best locate the MHU.
	(b) Have MHU staff engage in community committees and meetings to establish a community presence.
	(c) Establish and maintain routine communication and connection with community partners.
	(d) Create and maintain stable MHU schedules that accommodate patient needs.
Logistics: Electronic Health Record and Electronic Scheduling	(a) The MHU should establish its own wireless internet connection to avoid service disruption and potential privacy issues.
	(b) Using the practice’s electronic health record and electronic scheduling system helps ensure continuity of care, prevents errors, and allows for longitudinal tracking of health outcomes.
Logistics: Operational Oversight	(a) A high-functioning MHU program requires one or more program leaders with a broad project planning and project management skillset. This role will oversee and build fluency in operational processes such as: Building and maintaining schedules and visit templates Ensuring patient eligibility, billing, and entitlement screening Monitoring productivity, capacity, and staffing Overseeing staff training and professional development Managing team communication and meetings Deploying the MHU and negotiating policies like overnight parking and use of partners’ facilities and resources They will also develop a niche set of skills with respect to vehicle license and safety compliance, logbooks and safety checks, and logistics and immediate troubleshooting—sometimes across multiple MHUs simultaneously.
Logistics: MHU Team	(a) The MHU team should include a driver, provider, and clinical support staff (as needed), and an enabling services coordinator to assist patients with insurance enrollment, scheduling and coordination, access to social services, and to make sure they feel welcome at the MHU. Drivers can also serve as outreach workers or equivalent and provide transportation to laboratory services and pharmacies. A clinical staff member may be appointed to manage inventory and supplies and monitor compliance with clinical regulations.
	(b) MHU teams should prioritize hiring team members who share a geographic, social, and/or economic background with community members.
	(c) Providers who work on an MHU should be confident and competent in providing care for patients with a variety of health conditions, who are experiencing severe barriers to obtaining quality care, and who may have low levels of health literacy and patient activation.
	(d) MHU providers’ first priority should always be to address the immediate need of the patient that brought them to the MHU.
	(e) MHU providers should have the skills to offer and engage patients in chronic disease management (such as hypertension, diabetes, etc.) as well as preventive screenings and care.
	(f) MHU providers should also have the skills necessary to address the needs of patients in the community they are serving. These needs may include acute (non-emergent) conditions, substance use (including medications for substance use), mental health, HIV prevention and treatment, viral hepatitis care, wound care, and women’s health.
	(g) FQHCs should consider creating an internal “network” of MHU care providers for ongoing curbside consultation, support, and guidance on complex cases, or providing access to an experienced MHU provider to support real-time consultation and guidance.
	(h) The full MHU team should be well-versed in non-clinical necessities such as vehicle maintenance, personal and vehicular safety, HIPAA and Joint Commission compliance, billing, scheduling, and logistics, including situation-specific knowledge about parking, heating and cooling, snow removal/clearance, and safe use and removal of water, sewage, and medical waste from the MHU (as needed).
Addressing Healthcare Access Needs: Enabling Services	(a) Having dedicated staff for insurance enrollment and eligibility assistance ensures that financial barriers are removed. Assistance navigating through complex social systems or application processes helps build patients’ trust and incentivizes them to engage with the MHU.
Addressing Healthcare Access Needs: Social Setting	(a) Services can be delivered at familiar community locations such as libraries, community centers, food pantries, apartment complexes, churches, and shelter sites.
Addressing Healthcare Access Needs: Trust	(a) Having a team of staff dedicated to the MHU facilitates trust that is important to patients whose lives can be unstable. If one or two members of the team are always the same and the schedule is kept consistent then the MHU becomes a reliable foundation in the community or at the site regardless of what services are offered or if there is flexibility of some staffing.
Addressing Healthcare Access Needs: Consistent Care	(a) Forming a relationship with a primary care provider, care team member, or MHU staff member improves continuity of care. When MHUs use a rotating provider schedule, enabling services staff members can serve as the face of the program and provide additional stability.

Abbreviations: MHU: Mobile Health Unit; HIPAA: Health Insurance Portability and Accountability Act of 1996.

## Data Availability

The original contributions presented in this study are included in the article. Further inquiries can be directed to the corresponding author.

## Abbreviations

The following abbreviations are used in this manuscript:

U.S.	United States
MHU	Mobile health unit
FQHC	Federally Qualified Health Center
CHCI	Community Health Center, Inc.
CT	Connecticut
CKP	Center for Key Populations
ICD-10	International Classification of Diseases, 10th revision
HIV	Human immunodeficiency virus
STI	Sexually transmitted infection
HIPAA	Health Insurance Portability and Accountability Act of 1996
